# Depression, Sex and Gender Roles in Older Adult Populations: The International Mobility in Aging Study (IMIAS)

**DOI:** 10.1371/journal.pone.0146867

**Published:** 2016-01-15

**Authors:** Afshin Vafaei, Tamer Ahmed, Aline do N. Falcão Freire, Maria Victoria Zunzunegui, Ricardo O. Guerra

**Affiliations:** 1 Department of Public Health Sciences, Queen's University, Kingston, ON, Canada; 2 Département de médecine sociale et préventive, Université de Montréal, Montreal, QC, Canada; 3 Federal University of Rio Grande do Norte, Natal, RN, Brazil; Nathan Kline Institute and New York University School of Medicine, UNITED STATES

## Abstract

**Objectives:**

To assess the associations between gender roles and depression in older men and women and whether gender roles are independent risk factors for depression.

**Methods:**

International cross-sectional study of adults between 65 and 74 years old (n = 1,967). Depression was defined by a score of 16 or over in the Center for Epidemiologic Studies Depression Scale (CES-D). A validated 12-item Bem Sex Role Inventory (BSRI) was used to classify participants in gender roles (Masculine, Feminine, Androgynous, and Undifferentiated) using research site medians of femininity and masculinity as cut-off points. Poisson regressions were fitted to estimate the prevalence ratios (PR) of depression for each gender role compared to the masculine role, adjusting for sex, sufficiency of income, education, marital status, self-rated health, and chronic conditions.

**Results:**

Among men, 31.2% were androgynous, 26% were masculine, 14.4% were feminine, and 28.4% were undifferentiated; among women, the corresponding percentages were 32.7%, 14.9%, 27%, and 25.4%. Both in men and in women, depressive symptoms (CES-D≥16) were more prevalent in those endorsing the undifferentiated type, compared to masculine, feminine or androgynous groups. However, after adjusting for potential confounders, compared to the masculine group only those endorsing the androgynous role were 28% less likely to suffer from depression: PR of 0.72 (95% CI: 0.55–0.93). In fully adjusted models, prevalence rates of depression were not different from masculine participants in the two other gender groups of feminine and undifferentiated.

**Conclusions:**

Androgynous roles were associated with lower rates of depression in older adults, independently of being a man or a woman.

## Introduction

Mental health issues are worldwide problems imposing huge burdens on health care systems and lives of individuals with depression constituting a large proportion of mental disorders [1]. According to the World Health Organization 2004 statistics, globally, depression was the third most important cause of disease burden. Rates of depression vary considerably across the countries with a range in lifetime prevalence rates from approximately 3% in Japan to 16.9% in the United States. Most countries fall somewhere between 8 to 12 percent [2]. Rates of depression within a country are also not uniform. For example, it is estimated that lifetime prevalence of depression in Brazil is about 17% [3] but due to regional and cultural diversity, rates differ from 12.2% [4] and 19.5% [5] in the Southeast of Brazil to 37.5% [6] in the Northeast of Brazil.

What is universal is the sex differences; almost all over the world, depression is two to three times more common in women [2,3,7,8]. This differential burden also applies to other mental health issues. Prevalence of depression, anxiety and suicidal ideations is higher in women while men instead adopt hazardous health-related behaviors such as substance abuse, violent or antisocial behaviors and suicide [9]. Women are more exposed to stressful events and risk factors for depression during their life and may also react differently to those factors; two facts that contribute to explain women’s greater rates of depression [10–12]. It has been shown that mood regulation mechanisms in women tend to be emotion-focused, whereas in men the focus is more directly on solving the problem [13]. These sex differences in expression of and dealing with mental distress have been linked to the social roles favored by society for men and women [14]. The adoption of feminine roles by women may contribute to depression.

The strength of gender roles varies according to place, time, and cultural context. First, in many traditional societies, there is unequal power in marital relationships, favoring men’s autonomy and decision making power. Second, due to the division of labor, there are unequal distributions of parenthood and home care responsibilities with larger share of child care and homemaking duties for women and more out-of-home jobs for men under the accepted assumption that men’s primary role is to assure the family’s material need [15]. Lastly, there is a predefined emphasis on what is feminine and what is masculine in the society with practices that mainly promote the dominant social position of men and the subordinate social position of women [16]; a concept called by Connell: “hegemonic masculinity” [17]. Gender stratification is related to less power, lower social status, and fewer opportunities and resources for women which increases vulnerability to depression [18]. Restricted by gender roles, women are constrained to domestic tasks and have limited access to well-paid jobs and despite working full time are dependent on male members of the family, an inequality that may well extend to older ages and take different forms across cultures. We conceptualize gender roles as links between the gender structure of society (with mostly universal hegemonic masculinity) and personality. Personality traits, internalized throughout the life course by gender roles, lead to individual behaviours and then health outcomes. However, considering the complexity of the issue we adopted a narrow epidemiological focus and concentrated on direct health impacts of gender roles. Further investigations through the lens of this other literature are warranted.

Since the latter half of the twentieth century, societal gender roles attitudes have become relatively more egalitarian in more developed countries compared to less affluent ones [19]; however, the implications of these changes in attitudes and roles are not well understood in aging research and the impact they might have on patterns of psychological distress in older men and women has not been explored [20].

**Fig 1:**
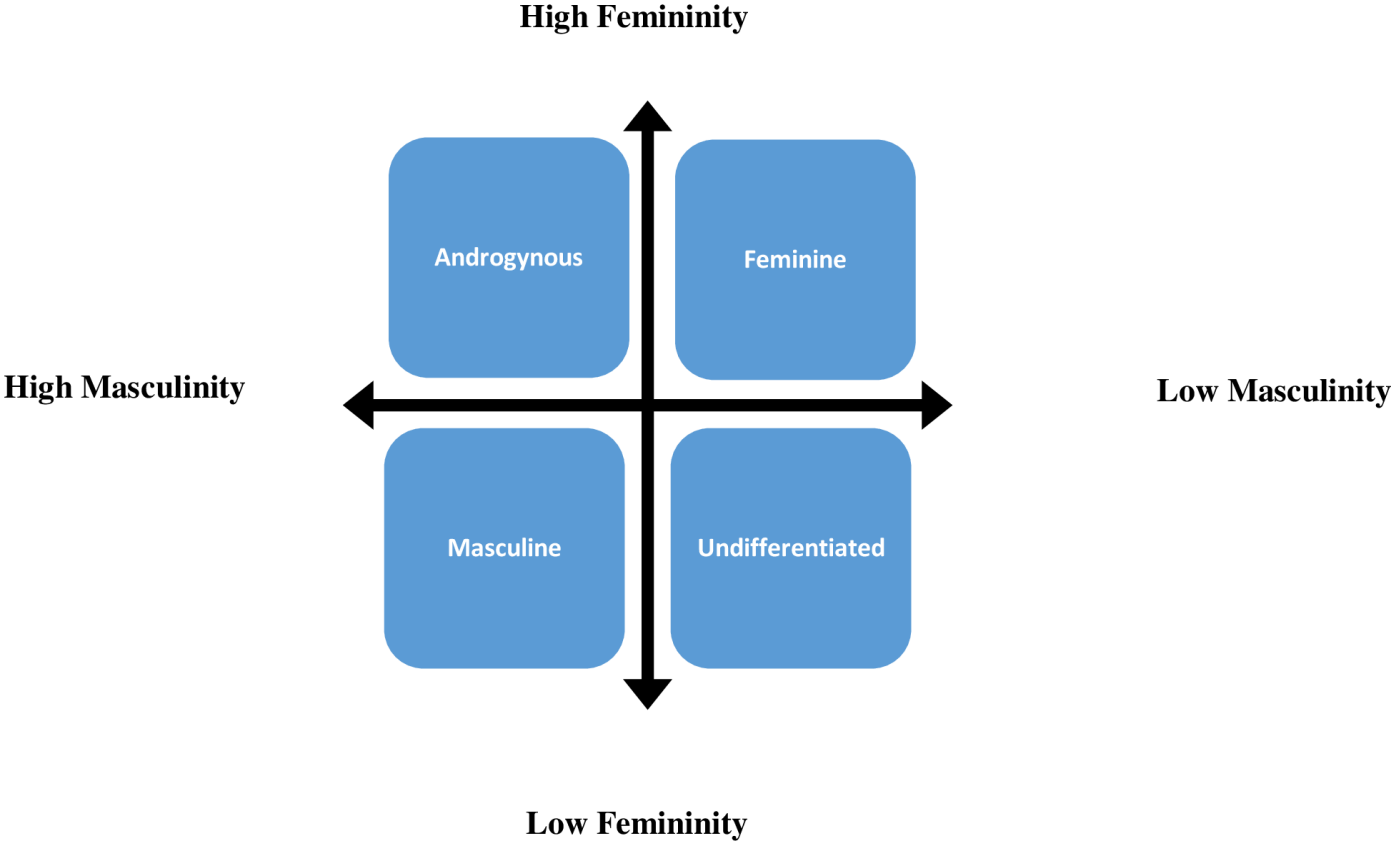
The Androgyny Model.

The documented fact that there are women who do not endorse femininity stereotypes and men who do not endorse masculinity stereotypes suggest that biological sex and gender roles are weakly related [21,22]. It has been suggested that masculinity and femininity are not the two opposite poles of a continuum; they co-exist and interact giving rise to gender roles with both masculine and feminine attributes. Bem was the first researcher who argued against the exclusive dichotomy of gender roles and defined four categories: masculine, feminine, androgynous and undifferentiated [23]. Based on her androgyny model (Fig 1), in addition to two generally accepted masculine and feminine gender roles, there are androgynous individuals who express both masculine and feminine traits and undifferentiated individuals who do not clearly endorse either masculine or feminine traits [24].

The gender role a person adopts has health impacts; generally the masculine gender role is associated with worse physical health [25], whereas the feminine gender role seems to be protective. Hunt et al [26] showed that the risk of death from coronary heart diseases is lower in men with feminine traits; however, femininity is related to worse mental health [27]. One potential reason for more depression in feminine individuals (either male or female) could be the passive responses they adopt in responding to life challenges [25] by focusing on personal concerns instead of adopting a problem solving approach. In contrast, androgynous people adopt a more interactive response. Depending on the situation they will mobilize their masculinity and will be assertive or use their femininity trait and show expressiveness and will be yielding. Flexibility in behavioral responses to stressful conditions results in being more adaptive and therefore more likely to enjoy better mental health [24]. This theoretical conceptualization has been supported by research. In adults, androgyny has been associated with better mental health [28] and fewer risky health behaviors [29]. Androgynous Spanish [21] and Canadian [30] older adults reported better self-rated physical health, life satisfaction, and mobility.

Most existing research on gender and aging has focused on health impacts of gender roles in separate populations of men [31,32] or women [33] or has looked into changes in gender roles as people age, not specifically on gender-health relationships [34,35]. The androgyny model for older adults is seldom considered. The main objective of the few studies [21,22,30] that have explored the androgyny models in older adults populations was validation of the gender role measures and not their health impacts. Research in this area is growing but in spite of widespread worldwide variations in gender equality, as demonstrated by the United Nations Gender Inequality Index [36], little international research has been conducted.

The International Mobility in Aging study (IMIAS) provides the opportunity to examine the associations between gender roles and depression in five international samples of older adults. We hypothesized that regardless of their living environment and biological sex, older adults endorsing the androgynous gender role will have lower prevalence of depression due to better psychological adaptation and higher competence. The objective of this study was to examine differences in prevalence of depression in older adults according to their gender roles. A secondary objective was to assess if gender roles constitute an independent risk in these older men and women.

## Methods

### Data sources/study populations

We obtained data from the International Mobility in Aging Study (IMIAS) [37]. This is a prospective study conducted in five cities: Kingston (Ontario, Canada), Saint-Hyacinthe (Quebec, Canada), Tirana (Albania), Manizales (Colombia), and Natal (Brazil). Questionnaires and instructions for measurement procedures were available in five languages (English, French, Albanian, Spanish, Portuguese) and were administrated by trained interviewers. For this study, we made use of baseline data collected in 2012.

The study population was composed of community—dwelling men and women aged 65–74 years. We aimed for 200 men and 200 women at each city and stratified the sample by sex. Due to restrictions imposed by Canadian ethics committees, hindering direct contact with potential participants, Canadian potential participants were invited by a letter from their primary care physician and asked to contact our field coordinator if they would like to participate in the study. To identify the potential participants, random samples were drawn from family practice lists of patients in the 65–74 age group. The family practices participating in the study came from all family medicine teams covering populations of Kingston and Saint-Hyacinthe. In Saint-Hyacinthe the sample was stratified by neighbourhood, while in Kingston due to ethical issues this stratification was not possible.

In Tirana, Manizales, and Natal, participants were randomly selected from the population in the 65–74 age group registered at neighbourhood health centres. Brazil and Albania have universal health coverage and practically all people in this age group are registered at the local primary care centres; however, in Colombia, the public healthcare network covers only approximately 70% of older adults.

Response rates were higher than 90% in Tirana, Manizales, and Natal. In Canadian cities, 95% of the subjects who contacted the field coordinator eventually participated in the study. Of a total 1,995 participants, 1,967 answered the Bem sex roles questionnaire and had complete data in all considered covariates and were included in the analysis.

### Ethics statement

The Research Centres of the University of Montreal (CR-CHUM), the Albanian Institute of Public Health, and the Research Ethics Boards of the Federal University of Rio Grande do Norte (Brazil), the general research ethics board of Queen’s University (Kingston) and University of Caldas (Columbia) approved the study. All study participants provided written informed consent and were told they could withdraw at any time.

### Measures

#### The outcome

Depression was assessed by the Center for Epidemiological Studies Depression Scale (CES-D) [38]. This scale is a screening tool comprised of 20 items related to depressive symptoms such as mood, somatic symptoms, interactions with others, and psychomotor functions and has been validated in French [39], Brazilian Portuguese [40], and Spanish [41] older adults populations as well as in southern and eastern Mediterranean regions [42,43] and low income settings [44]. The frequency of each symptom in the week prior to interview were scored according to the frequency of symptoms (0 = never or rarely, 1 = sometimes, 2 = often, and 3 = most of the time, or always) which gives a final score in a range between 0 to 60 points. We utilized the established cut-off point of 16 as suggestive for depression [38].

#### Gender roles

We used the Bem Sex Role Inventory (BRSI) to measure gender roles [23]. BSRI measures participants’ instrumental (masculine) and expressive (feminine) traits and thereby their gender roles and appears to be valid across geographical and cultural contexts [21,22]. As per precedents [21,22], we used a short version of the BSRI composed of 12 items. Using Likert-like responses with seven options, participants were asked to rate from ‘never true’ to ‘almost always true’ if they perceive themselves as gentle, warm, tender, sympathetic, affectionate, and are sensitive to others’ needs (original feminine items) and also whether they think they possess leadership abilities, strong personality and also if they act like a leader, defend own beliefs, make decisions easily, and are dominant (original masculine items). The validity and internal reliability of this short version of the BSRI in IMIAS participants have been examined via confirmatory factor analysis and showed to be high. The details of psychometric properties of BSRI items and the full validation methodology have been described in a separate paper under review [45].

The common classification method of median split [46] was used to categorize the study population into four gender role groups as per existing precedents [21,22,30]. First, the median of distributions of masculinity and femininity scales were established. Then femininity and masculinity scores were compared to the median. If the individual’s score was below the median on both the feminine and masculine scales, the person was classified as ‘undifferentiated’. If the scores on both the femininity and masculinity scales were equal to or above the median, that individual was classified as ‘androgynous’. Those people who were equal to or higher than the median on the feminine scale and lower on the masculine scale were classified as ‘feminine’. Finally, those who were equal to or higher than the median on the masculine scale and lower on the feminine scale were classified as ‘masculine’. The distributions of the masculinity and femininity scores were statistically different across research cities, thus the reliability of results could be influenced by culturally derived differences. In order to control for these differences across cities, city-specific medians of the distributions of femininity and masculinity were used as cut-off points.

#### Covariates

Factors with potential confounding effects on the relationship between gender roles and depression were included in multivariate analyses. Women consistently report more depression symptoms and the probability that a woman expresses feminine gender role is higher, therefore biological sex was the most important potential confounder.

Age, marital status, education and income are documented risk factors for depression in older adults [10] and they could also be associated with gender roles. Education was assessed based on the highest level of schooling completed by participants. Responses were grouped into three categories: less than secondary school, secondary, and post-secondary. Income was assessed by asking the question, “Is your monthly income sufficient to cover basic needs?” The answers were divided into three categories: very sufficient, barely sufficient, and insufficient. Self-reported health (SRH) which has been used extensively as an indicator of general health [30,47,48] was used for the same purpose in this study. SRH has been shown to be a good predictor of mortality in older adults [49,50]. Our measure of SRH encompassed four categories: ‘very good’, ‘good’, ‘fair’, and ‘bad’. In the analysis, ‘very good’ and ‘good’ answers were combined to create a *good* SRH group and other options were grouped as *poor* SRH. The number of self-reported chronic conditions has been also used as an indicator of health status and a potential confounder [10].

### Statistical analysis

To define the four Bem sex roles types we used the femininity and masculinity scores obtained from confirmatory factor analyses of the BSRI and applied city-specific median based cutoffs as explained above. Distributions of all variables in total and across sex and gender roles groups were estimated and differences were statistically examined using Chi-square and ANOVA tests where appropriate. We used Poisson regressions with robust variance to estimate the prevalence ratios (PR) of depression in different gender role groups (masculine role as the reference category) adjusting for sex and all other potential confounders [51,52]. Multiplicative product terms between gender roles and biological sex as well as research sites were used to test for interactions at a statistical significance level of p<0.05. All statistical analyses were conducted with SPSS v21.0.

## Results

Participants consisted of 942 men and 1,025 women with an average age of 69.1 (SD: 2.9). More than half of the participants (64.6%) were married and women were significantly more single or widowed (p<0.001). About one third had less than high school education and 36% of respondents reported insufficient income to cover their basic needs. The samples were relatively healthy; 56% reported to be in good or very good health and 43% had zero or only one chronic condition (Table 1). In 379 (19.4%) of participants, the responses to the CES-D questions were suggestive of depression (CES-D≥16). The prevalence of depression was higher in women than in men (26.1; 95% CI: 23.6–29.0) vs. (12.0; 95% CI: 9.9–14.1) producing a significant women/men prevalence ratio of 2.2 (95% CI: 1.8–2.7).

**Table 1:** Distribution of demographic and socioeconomic and health indicators.

	Men (n, %)	Women (n, %)	Total (n, %)	p-value*
**Age (n, mean, (SD))**				
	942, 9.1, (2.9)	1,025, 69.1, (2.8)	1,967, 9.1, (2.8)	0.797
**Marital status**				<0.001
Single	41 (4.4)	79(7.7)	120 (6.1)	
Married	754 (80.0)	517 (50.4)	1,271(64.6)	
Widowed	44 (4.9)	281 (27.4)	325 (16.5)	
Divorced	103(10.7)	148 (14.4)	251(12.8)	
**Education**				0.013
Less than secondary	300 (31.8)	376 (36.7)	676 (36.7)	
Secondary	232 (24.6)	268 (26.1)	500 (25.4)	
More than secondary	410 (43.5)	381(37.2)	791(40.2)	
**Income sufficiency**				0.013
Very sufficient	312 (33.1)	284 (27.7)	596 (33.2)	
Barely sufficient	317 (33.7)	344 (33.7)	659 (38.6)	
Insufficient	313 (33.2)	388 (38.6)	695 (36.0)	
**Self-rated health**				<0.001
Very good, good	568 (60.4)	535 (52.2)	1,103 (56.1)	
Fair, bad	373 (39.2)	489 (47.8)	862 (43.9)	
**Chronic Conditions**				<0.001
0–1	485 (51.5)	358 (34.9)	843 (42.9)	
2–3	388 (41.2)	506 (49.4)	894 (45.4)	
4 and more	69 (7.3)	161 (15.7)	230 (11.7)	
**Depression**				<0.001
CES-D ≥ 16	116 (12.3)	268 (26.1)	384 (19.5)	
CES-D <16	826 (87.7)	757 (73.9)	1,583 (80.5)	

* From Chi-square for comparing frequencies and *t*-test for comparing means

Numbers in parenthesis represent column percentages

The frequency of gender roles varied across research cities and there were associations between gender role and sex in all cities except in Natal. However, although men tended to endorse masculinity more than women and women tended to endorse femininity more than men, high proportions of both men and women considered themselves androgynous or undifferentiated. About 20% of men endorsed femininity while a similar proportion of women endorsed masculinity (Table 2).

**Table 2:** Endorsement of Gender Roles by older men and women by research city.

**Gender roles**	Total n, (%)	Men n, (%)	Women n, (%)
Kingston			
Masculine	67 (17.0)	38 (20.7)	29 (13.9)
Feminine	80 (20.4)	26 (14.1)	54 (25.8)
Androgynous	140 (35.6)	57 (31.0)	83 (39.7)
Undifferentiated	106 (27.0)	63 (34.2)	43 (20.6)
Total	393 (100)	184 (100)	209 (100)
Saint Hyacinthe			
Masculine	82 (20.9)	48 (25.5)	34 (16.7)
Feminine	91 (23.2)	32 (17.0)	59 (28.9)
Androgynous	122 (31.1)	66 (35.1)	56 (27.5)
Undifferentiated	97 (24.7)	42 (22.3)	55 (27.0)
Total	392 (100)	188 (100)	204 (100)
Tirana			
Masculine	85 (22.0)	60 (32.8)	25 (12.3)
Feminine	85 (22.0)	23(12.6)	62 (30.4)
Androgynous	110 (28.4)	51 (27.9)	59 (28.9)
Undifferentiated	107 (27.6)	49 (26.8)	58 (28.4)
Total	387 (100)	183 (100)	204 (100)
Manizales			
Masculine	90 (22.9)	57 (29.2)	33 (16.7)
Feminine	84 (21.4)	28 (14.4)	56 (28.3)
Androgynous	114 (29.0)	49 (25.1)	65 (32.8)
Undifferentiated	105 (26.7)	61 (31.3)	44 (22.2)
Total	393 (100)	195 (100)	198 (100)
Natal			
Masculine	74 (18.4)	42 (21.9)	32 (15.2)
Feminine	73 (18.2)	27 (14.1)	46 (21.9)
Androgynous	143 (35.6)	71 (37.0)	72 (34.3)
Undifferentiated	112 (27.9)	52 (27.1)	60 (28.6)
Total	402 (100)	192 (100)	210 (100)
The whole sample			
Masculine	398 (20.2)	245 (26)	153 (14.9)
Feminine	413 (21)	136 (14.4)	277 (27)
Androgynous	629 (32)	294 (31.2)	335 (32.7)
Undifferentiated	527 (26.8)	267 (28.4)	260 (25.4)
Total	1,967 (100)	942 (100)	1,025 (100)

Note: Chi-square p-value for the relationship between gender roles and sex P <0.001 in Kingston, Tirana and Manizales; 0.006 in Saint-Hyacinthe; and p = 0.11 in Natal

**Fig 2:**
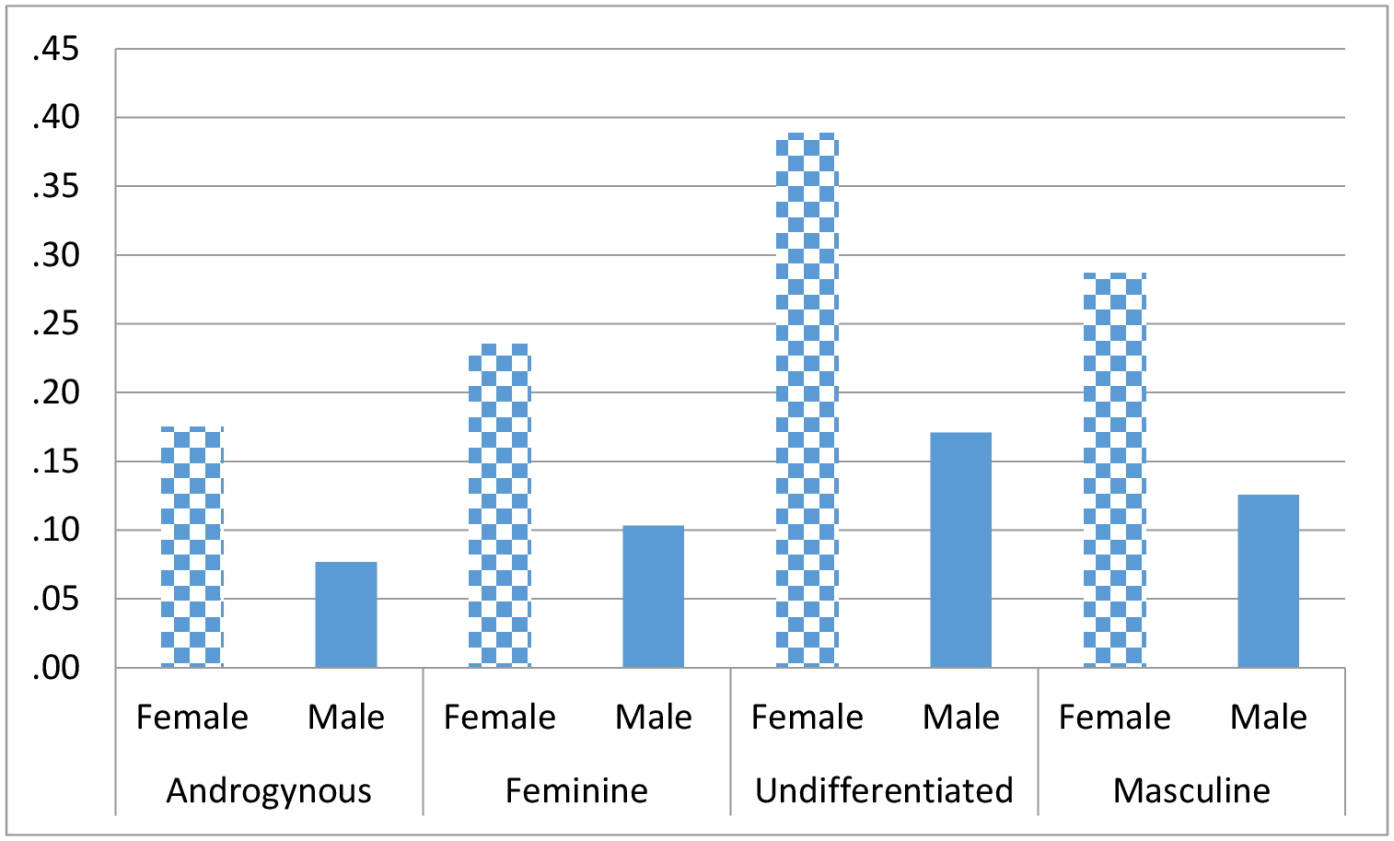
Depression prevalence by Bem Sex Roles in men and women.

Sex-specific bivariate analyses suggested that depression might be related to perceived gender roles. The estimated sex specific prevalence rates of depression by gender roles are shown in Table 3 and illustrated graphically in Fig 2. Both in men and in women, depression prevalence was the lowest among androgynous people and the highest among undifferentiated participants. Prevalence of depression for those endorsing masculine and feminine roles were in-between of androgynous and undifferentiated groups.

**Table 3:** Depression prevalence (CES-D ≥16) according to Bem sex roles in men and women.

Bem sex roles	Sex	Prevalence (95% CI)
Masculine	Women	29% (22%-37%)
	Men	13% (8%-17%)
Feminine	Women	23% (18%-28%)
	Men	11% (6%-16%)
Androgynous	Women	18% (14%-22%)
	Men	7% (4%-10%)
Undifferentiated	Women	38% (32%-44%)
	Men	18% (13%-23%)
The whole sample	Women	26% (23%-29%)
	Men	13% (11%-15%)

Gender roles were also significantly associated with all the examined indicators of socioeconomic status and perceptions of health (Table 4).

**Table 4:** Distribution of socio-demographic and health indicators by gender roles.

	Masculine	Feminine	Androgynous	Undifferentiated	p-value*
**Age (mean, SD)**					0.680
	69.1 (2.9)	69.2 (2.9)	69.1 (2.9)	69.0 (2.8)	
**Sex** (n, %)					<0.001
Men	245 (26.0)	136 (14.4)	294 (31.2)	267 (28.3)	
Women	153 (14.9)	277 (27.0)	335 (32.7)	260 (25.4)	
**Marital status** (n, %)					0.002
Single	23 (19.2)	25 (21.4)	31 (25.8)	41 (34.2)	
Married	283 (22.3)	245 (19.3)	399 (31.4)	344 (27.1)	
Widowed	52 (15.0)	92 (28.8)	110 (33.8)	71 (21.8)	
Divorced	40 (16.0)	51 (20.4)	89 (35.5)	71 (28.3)	
**Education** (n, %)					0.007
Less than secondary	138 (20.4)	149 (22.4)	190 (28.1)	199 (29.4)	
Secondary	91 (18.2)	110 (22.1)	153 (30.6)	146 (29.2)	
More than secondary	169 (21.4)	154 (19.5)	286 (36.2)	182 (23.0)	
**Income sufficiency** (n, %)					<0.001
Very sufficient	138 (23.2)	116 (19.5)	215 (36.1)	127 (21.3)	
Barely sufficient	107 (16.1)	155 (23.5)	209 (31.6)	191 (28.9)	
Insufficient	153 (21.6)	142 (20.3)	205 (28.9)	209 (29.5)	
**Self-rated health** (n, %)					<0.001
Very good, good	221 (20.0)	222 (20.1)	402 (36.4)	258 (23.4)	
Fair, bad	176 (20.4)	191 (22.4)	226 (26.2)	269 (31.2)	
**Chronic conditions** (n, %)					0.057
0–1	185 (21.8)	168 (20.0)	267 (31.7)	223 (26.5)	
2–3	166 (18.6)	195 (22.0)	305 (34.1)	228 (25.5)	
4 and more	47 (20.4)	50 (21.7)	57 (24.8)	76 (33.0)	

*From Chi-square for comparing frequencies and *t*-test for comparing means

Results of multivariate analyses of associations between gender roles and depression are presented in Table 5. Keeping the masculine category as the reference group, in unadjusted models, prevalence rates of depression in undifferentiated participants were 1.5 times higher than those with masculine gender role (Prevalence ratio (PR) = 1.47; 95% CI: 1.15–1.88)), whereas the relative difference was lower for the androgynous group (Prevalence ratio (PR) = 0.67; 95% CI: 0.51–0.90)). Only those with the feminine gender role were not statistically different from the masculine type. Adjustment for biological sex did not change the results of bivariate analyses. In fact the prevalence ratio of depression of women compared with men after controlling for gender roles remained practically unaltered from the unadjusted estimate of 2.19 (95% CI 1.79–2.68) to the 2.22 (95% CI 1.82–2.71). Contrary to expectations, the association between sex and depression was independent of gender roles.

**Table 5:** Prevalence rate ratios for depression.

	Prevalence ratio (95%CI)		
	Unadjusted	Adjusted by sex	Adjusted by all covariates
**Gender roles**			
Undifferentiated	1.47 (1.15–1.88)	1.35 (1.06–1.71)	1.22 (0.98–1.52)
Feminine	1.00 (0.75–1.30)	0.81 (0.61–1.07)	0.83 (0.64–1.07)
Androgynous	0.67 (0.51–0.90)	0.60 (0.45–0.80)	0.72 (0.55–0.93)
Masculine (reference)	-	-	-
**Sex**			
Women vs. men	2.19 (1.72–2.68)	2.22 (1.82–2.71)*	1.64 (1.34–2.00)
**Marital status**			
Single vs. married			1.47 (1.11–1.94)
Widowed vs. married			1.37 (1.13–1.67)
Divorced vs. married			1.11 (0.85–1.45)
**Education**			
Less than secondary vs. more than secondary			1.13 (0.87–1.46)
Secondary vs. more than secondary			1.09 (0.88–1.34)
**Income sufficiency**			
Insufficient vs. very sufficient			1.41 (1.05–1.90)
Barely sufficient vs. very sufficient			1.26 (0.95–1.67)
**Self-rated health**			
Poor vs. good health			4.41 (3.37–5.78)
**Chronic conditions**			
More than 3 vs. 0 or 1			1.66 (1.23–2.13)
2 or 3 vs. 0 or 1			1.31 (1.05–1.64)

Note: Prevalence rate ratios were estimated directly by Poisson regressions with robust variance

*Adjusted for gender roles

After adjustment for all potential confounders, compared to masculine participants, prevalence rates of depression were 28% lower (PR = 0.72; 95% CI: 0.55–0.93) in those categorized as androgynous. The significant excess risk for depression in participant with undifferentiated gender roles disappeared after adjustment for confounders, although close to significant confidence interval of (0.98–1.52) suggests a possible association. Prevalence rates of depression in feminine and masculine groups remained non-significantly different in fully adjusted models (PR = 0.83; 95% CI: 0.64–1.07). All tested interactions between biological sex, cities, and gender roles were not significant, demonstrating that the association between gender roles and depression is not modified by biological sex and research sites. To explore the potential cultural differences in the relationship of interest we also divided the data into three regions: Canada, Latin America, and Albania and re-performed the analysis. Again, in theses stratified analyses no changes were observed in the significance of associations between gender roles and depression (data not shown, available upon request).

Other factors with independent effects on rates of depression were being widowed or single, income insufficiency, poor SRH, and having two or more chronic diseases. Age was not related to depression or to gender roles and thus it was not included in the regression models.

To assess if our results were sensitive to the use of city-specific masculinity and femininity median cut-off points used to define the gender roles types, we carried out a sensitivity analysis. We classified the subjects into the four gender roles groups using a common cut-off point defined by the medians of the distributions of masculinity and femininity in the pooled sample. We then constructed multiple regression models with these newly built gender groups and entered ‘city’ into models as a potential confounder. Results were very similar and are available upon request from authors.

## Discussion

We demonstrated that both in men and in women, gender roles were related to depression and this association is independent of sex, even after extensive adjustment for socioeconomic and health status variables. In addition, the strength of the association between sex and depression does not vary after adjustment for gender roles, indicating that both gender roles and sex impact depression independently. In this study of relationships between gender roles and depression in older adults, we made use of five diverse samples of older adults from an international study. To account for differences in perception of gender roles in different settings we employed the novel approach of using city-specific cut-off points and used within-city validated measures of gender roles [45].

Our results were generally consistent with the ‘androgyny model’ [21,24,28,29] which suggests individuals with both masculine and feminine traits have more flexibility in coping with stressful life events and hence are mentally healthier. We observed that androgynous older men and women regardless of their place of residence reported the fewest depression symptoms. Another study [32] in adult African American males reported similar findings of lowest rates of depression in androgynous individuals compared to other three groups. No similar study has been conducted in older adults.

It has been generally shown that femininity increases the risk for depression [12]; surprisingly, in this study the prevalence rate of depression was relatively high in those with masculinity traits. Masculine individuals in the pursuit of power and reaching to masculine hegemony may harm themselves physically [53] and it is possible that older individuals because of their masculinity traits, cannot adapt well to the aging-related physical and social power loss. The stress and disappointment associated with this maladaptation process maybe a contributive factor to the occurrence of depression. There are some evidence suggesting depressive symptoms increase as people age but at a higher rate in men than in women [54]; however, this remains a theory to be explored in future studies.

Apart from the ‘androgyny model’ theory, the differences in the prevalence of depression across gender roles can also be due to gender inequalities in access to socioeconomic resources and opportunities [11]. Two hypotheses have been suggested to explain how social factors relate to gender differences in health. The differential exposure hypothesis suggests that higher prevalence rates of depression in women are due to more frequent exposures to cumulative social and economic disadvantages while the differential vulnerability hypothesis proposes that relative to men, the harmful impacts of social disadvantages are stronger in women [10].

Contrary to other studies in older adults [21,22], in all cities except Natal, biological sex was related to gender roles with significantly more feminine traits in women and more masculine traits among men. However, because the majority of participants identified themselves as androgynous or undifferentiated, our results still provide evidence against dichotomy of gender roles.

We were aware of the fact that the meaning of gender roles may not be the same for participants from Canada, Colombia, Brazil, and Albania. These countries are very different culturally and economically. More importantly, according to the 2014 Human Development Report of United Nations, Gender Inequality Index varies dramatically between these countries; among 187 countries, Canada ranks 33, Albania 44, Brazil 85, and Colombia 92. As explained above, in the main analysis to control for gender inequalities across cities we used city-specific medians of femininity and masculinity scores which produced similar results to those obtained from the pooled sample medians. This suggests that the association between gender roles and depression is robust across cultures.

Our study has strengths because it is based on a solid gender theory. The androgyny model of gender roles has been extensively discussed theoretically and has been used in several epidemiological studies. We used psychometrically assessed gender role measures and directly estimated prevalence ratios by constructing Poisson models with robust estimates, as suggested by the most recent methodological references [52]. To our best knowledge, our study was the first that explored the relationships between gender roles and depression in older adults using an international database. We showed that similar to younger people, gender role is associated with depression in older adults and this finding is consistent across very different cultures.

We also recognize that our study has some limitations. We used validated measures for the main variables of gender roles and depression; however, due to the self-report nature of data the possibility of information bias cannot be ruled out. Cross-sectional data preclude establishment of the temporal aspect of causality and even reverse causality is a possibility in this relationship. BSRI items mostly include positive personality traits [23] and those who are depressed may be less likely to score themselves high on any measure of positive traits and ending up as 'undifferentiated'. Future longitudinal studies can provide a more definite answer for this limitation.

## Conclusion

Male sex as well as androgynous gender role were independent protective factors for depression in older adults. The unexpected higher prevalence of depression observed in masculine types may be due to age-related loss of power and control.

Further studies are needed to determine the universality of our findings in other populations. These studies may include researchers from other disciplines with different and possibly wider theorizations.
